# Non‐coding RNAs in cardiomyocyte proliferation and cardiac regeneration: Dissecting their therapeutic values

**DOI:** 10.1111/jcmm.16300

**Published:** 2021-01-25

**Authors:** Xiaoxuan Dong, Xiuyun Dong, Feng Gao, Ning Liu, Tian Liang, Feng Zhang, Xuyang Fu, Linbin Pu, Jinghai Chen

**Affiliations:** ^1^ Department of Cardiology Provincial Key Lab of Cardiovascular Research Second Affiliated Hospital Institute of Translational Medicine Zhejiang University School of Medicine Hangzhou China; ^2^ Department of Cardiology Shanxi Cardiovascular Hospital Taiyuan China

**Keywords:** cardiomyocyte proliferation, gene therapy, non‐coding RNAs

## Abstract

Cardiovascular diseases are associated with high incidence and mortality, contribute to disability and place a heavy economic burden on countries worldwide. Stimulating endogenous cardiomyocyte proliferation and regeneration has been considering as a key to repair the injured heart caused by ischaemia. Emerging evidence has proved that non‐coding RNAs participate in cardiac proliferation and regeneration. In this review, we focus on the observation and mechanism that microRNAs (or miRNAs), long non‐coding RNAs (or lncRNAs) and circular RNA (or circRNAs) regulate cardiomyocyte proliferation and regeneration to repair a damaged heart. Furthermore, we highlight the potential therapeutic role of some non‐coding RNAs used in stimulating CMs proliferation. Finally, perspective on the development of non‐coding RNAs therapy in cardiac regeneration is presented.

## 1 INTRODUCTION

Adult mammalian cardiomyocytes (CMs) were considered differentiated cells and unable to proliferate. Foetal CMs proliferate during development but lose this ability rapidly after birth. The myocardium undergoes a transition from hyperplastic to hypertrophic growth shortly after birth. Following this transition, the main growth pattern is an increase in cell size and myofibril density, rather than in cell number.^1^ Recently, researchers retroactively analysed the integration of ^14^C isotopes into human cardiomyocytes who lived during the atmospheric nuclear bomb testing conducted from the early 1950s to 1963.^2^ Using carbon dating techniques and mathematical modelling, the estimated renewal rate of human CMs after birth is nearly 1% per year.^2^ However, as one grows older, the renewal rate decreases to 0.3%.^2^ Another study showed that CM proliferation contributes to developmental heart growth in young humans, indicating children and adolescents may be able to regenerate in heart diseases.^3^ More importantly, increasing studies reached the consensus that the regenerated adult CMs are from proliferation of pre‐existing CMs but not endogenous progenitor cells.^4^, ^5^ Although adult CMs can divide into two cells, these events naturally occur at a very low rate, which is not sufficient to restore the heart function after injury. Because of the dramatic decline in CMs cycle re‐entry activity and the loss of regeneration potential in adult hearts, there has been increasing research interest to understand the cellular mechanism of CMs division. Numerous studies have reported that in CMs, signals from growth factors, internal signalling pathways, microRNAs and cell cycle regulators can promote the cell cycle re‐entry in injured hearts.^6^, ^7^, ^8^, ^9^

**FIGURE 1:**
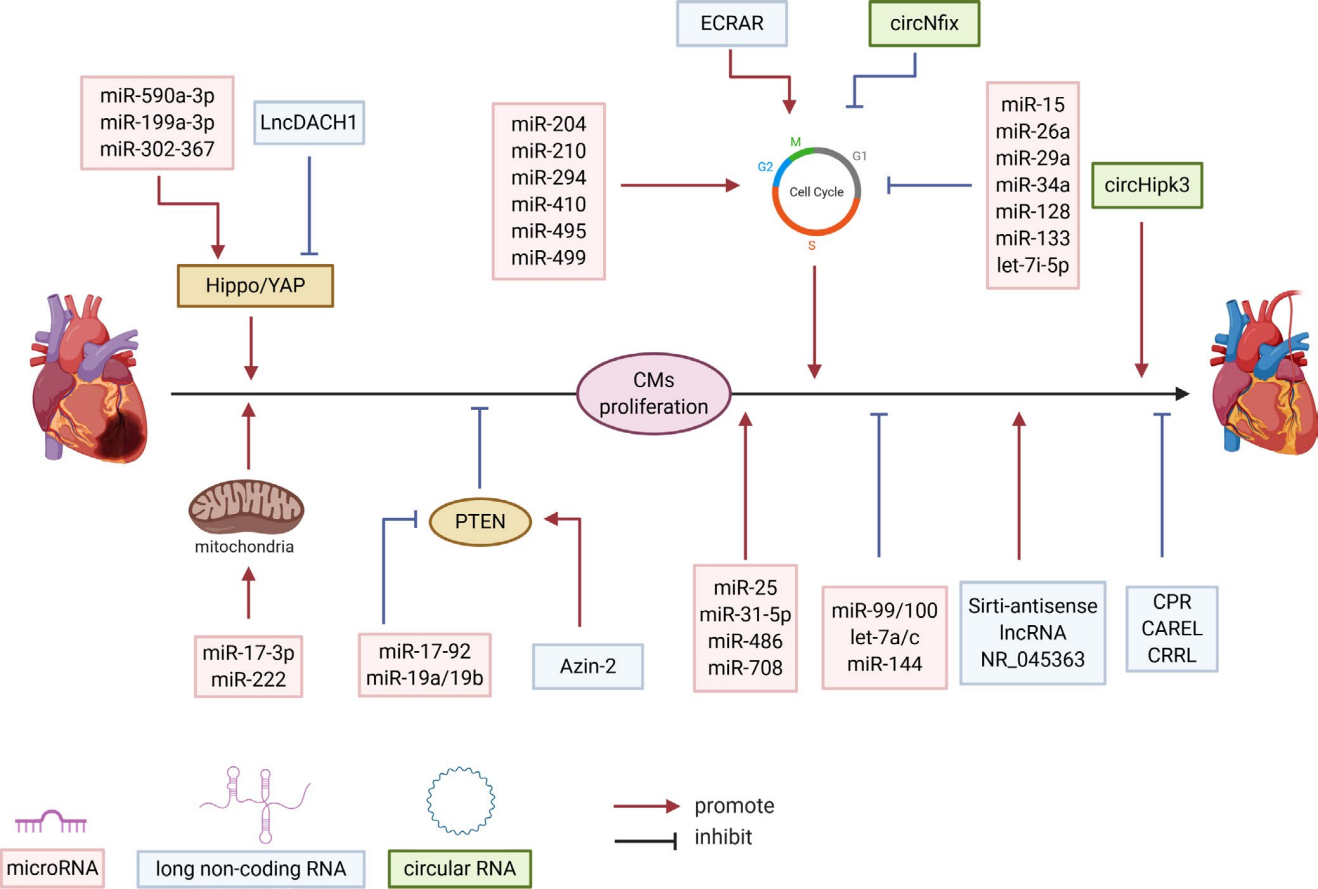
miRNAs, lncRNAs and circRNAs regulate cardiomyocyte proliferation to promote heart regeneration after injury. MicroRNAs, lncRNAs and circRNA are represented by red, blue and green boxes, respectively. The arrow indicates the promoting effect and the blunt end arrow indicates the inhibiting effect. (Created with BioRender.com)

**FIGURE 2:**
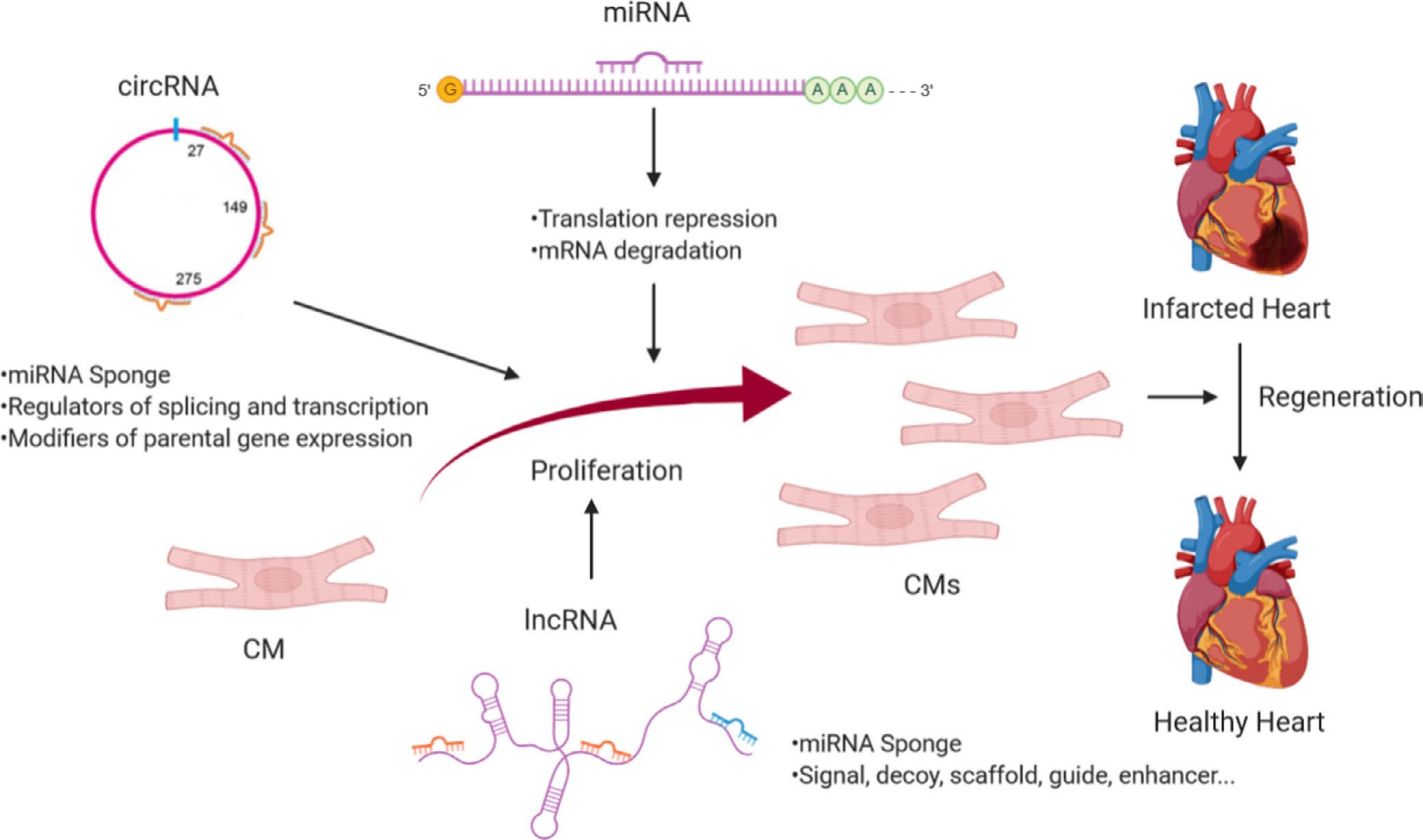
General mechanisms of ncRNAs in CM proliferation and heart regeneration. miRNAs recognize their targets mRNAs by “seed region” resulting in translation inhibition and protein degradation. CircRNAs and lncRNAs could both act as a sponge to compete with endogenous miRNAs. LncRNAs function as signal, decoy, scaffold, guide and enhancer depends on their specific subcellular location. CircRNAs exert their biological function as regulators of splicing and transcription and modifiers of parental gene expression. These different kinds of non‐coding RNAs regulate CM proliferation through their downstream targets to form complex signal pathways. (Created with BioRender.com)

Protein‐coding RNAs account for less than 2% of all of the transcribed RNAs.^10^ A large body of evidence has shown that non‐coding RNAs play important roles in biological processes and diseases. Based on the lengths, non‐coding RNAs can be subdivided into 2 major groups: (a) small non‐coding RNAs (<200 nucleotides) including rRNA, tRNA, microRNAs, PIWI‐interacting RNAs and endogenous short interfering RNAs, etc and (b) long non‐coding RNAs that have transcripts larger than 200 nucleotides in length and have no known protein‐coding function.^11^, ^12^, ^13^ In this review, we focus on non‐coding RNAs’ regulatory and therapeutic roles in CMs proliferation and cardiac regeneration (Figure 1). Besides, the general mechanism of ncRNAs in CM proliferation and heart regeneration is depicted in Figure 2.

## 2 NON‐CODING RNAS REGULATE CMS PROLIFERATION

### 2.1 miRNAs regulate CMs proliferation

In the cardiovascular system, miRNAs perform their physiological and pathological function in cardiac development,^14^, ^15^ diseases^16^, ^17^ and regeneration.^18^, ^19^ Here, we discuss the roles of miRNAs on CMs proliferation and the associated mechanism, which are summarized in Table 1.

**TABLE 1:** microRNAs regulate cardiomyocytes proliferation

microRNAs	Spices	In vitro	In vivo			Mechanism	Ref
			Animal model	Operation	Stage		
*Pro‐proliferation microRNAs*							
miR‐199a miR‐590a	Mice Rat	NMVMs NRVMs AMVMs	WT mice	injected synthetic miRNAs to neonatal rats injected AAV9‐hsa‐miR‐590, ‐199a after MI injury	Neonatal Adult	hsa‐miR‐590‐3p and hsa‐miR‐199a‐3p directly regulate ***Homer1*** and ***Hopx*** to promote CMs proliferation whereas ***Clic5*** is a direct target of the former miRNA only	20
						miR‐199a‐3p directly targets **TAOK1**, **β‐TrCP**, but not STK38L,to promote CMs proliferation by activating the YAP signal pathway miR‐199a‐3p also impedes filamentous actin depolymerization by targeting **Cofilin2**, a process activates YAP nuclear translocation	22
miR‐17‐92 cluster	Mice Rat	NRVMs AMVMs	miR‐17‐92^fl/fl^; Nkx2.5‐Cre miR‐17‐92^TG/TG^; Nkx2.5‐Cre miR‐17‐92^TG/TG^; α‐MHC‐Cre miR‐17‐92^TG/TG^; α‐MHC‐MCM	MI injury	Embryonic neonatal adult	miR‐17‐92 cluster targets **PTEN** to induce CMs proliferation	41
miR‐19a/19b	Mice	NRVMs AMVMs	WT mice	Injected miR‐19a/b mimics after MI injury	Adult	miR‐19 directly targets **PTEN** to induce CMs proliferation, **Bim** to inhibit apoptosis and **SOCS1** to suppress immune response	42
miR‐25	Rat	NRVMs HVCMs	‐	‐	‐	miR‐25 targets **Bim** to induce CMs proliferation and migration	43
miR‐302/367	Mice	NMVMs	miR‐302‐367^fl/fl^; Nkx2.5‐Cre R26R‐miR302‐367^Tg/+^; Nkx2.5‐Cre R26R‐miR302‐367^Tg/+^; Myh6‐MCM	MI injury	Embryonic neonatal Adult	miR302‐367 targets **Mst1**, **Lats2** and **Mob1b**, components of the Hippo signal transduction pathway to promote CMs proliferation	23
		NMVMs	WT mice Myh6‐MCM; R26R‐Confetti	Intracardiac injection of Gel‐miR‐302 MI injury	Neonatal adult		24
miR‐17‐3p	Mice Rat	NRVMs	Exercise mice model	Antagomir tail‐vein injection	Neonatal Adult	miR‐17‐3p directly targets **TIMP3** to enhance CMs proliferation and indirectly regulates **PTEN** to promote CMs hypertrophy	36
miR‐222	Mice	NRVMs	Exercise mice model Tg‐miR‐222 mice	I/R injury	Neonatal Adult	miR‐222 inhibits **p27** and **HIPK1** to exert the proliferative effects in cultured CMs, while reduces **Hmbox‐1** to promote cellular hypertrophy	38, 40
miR‐31a‐5p	Rat	NRVMs	WT rats	Intraperitoneally injection miR‐31a‐5p antagomir	Neonatal	miR‐31a‐5p targets **RhoBTB1** to mediate the regulatory effect of CMs proliferation	44
miR‐204	Mice Rat	NRVMs ARVMs	β‐MHC‐miR‐204 precursor	‐	Embryonic neonatal adult	miRNA‐204 mediates the proliferative growth of CMs by down‐regulation **Jarid2**	28
miR‐210	mice rat	ARVMs	miR‐210 TG	I/R injury MI injury	Adult	miR‐210 directly targets the cell cycle inhibitor **APC** to stimulate CMs proliferation	31
miR‐294	Mice	NRVMs AFVMs	WT mice	MI injury	Neonatal Adult	miR‐294 blunts **Wee1** leading to increased activity of the cyclin B1/CDK1 complex and improved CMs proliferation	32
miR‐410, miR‐495	Mice Rat	NRVMs	MEF2A KO mice	‐	Neonatal	miR‐410 and miR‐495 induce CMs proliferation by directly inhibiting the coactivator **Cited2**	33
miR‐486	Mice Sheep	EMCMs	WT mice	Injected miR‐486 mimics to neonatal mice	Neonatal	miR‐486 indirectly decreases **FoxO1** and **Smad** signalling meanwhile increased **Stat1** expression level who associated with Gata4 and SRF to stimulate CMs proliferation	46
miR‐497	Mice	‐	WT mice	Injected with miR‐497 agomir before I/R injury	Adult	miR‐497 promotes CMs proliferation and inhibits apoptosis through targeting **Mfn2**	93
miR‐499	Mice Rat	P19CL6 NRVMs HeLa	‐	Stable cell lines	Neonatal	miR‐499 stimulates the proliferation and inhibits apoptosis of P19CL6 cells in the late stage of cardiac differentiation via its effects on **Sox6** and **cyclin D1**	34
miR‐708	Mice Rat	H9c2 NMVMs NRVMs	WT mice	Isoproterenol induced heart injury treated with miR‐708 mimics	Neonatal Adult	miR‐708's pro‐proliferation effect is dependent at least partly on the inhibiting expression of **Mapk14**	87
miR‐1825	Rat	hiPS‐CMs ARVMs	WT mice	Injected AAV‐miR‐1825 to neonatal mice	Neonatal Adult	● miR‐1825 impedes mitochondrial mass and function by directly inhibiting **NDUFA10** ● miR‐1825 inhibits cell cycle genes **Rb1** and **Meis2** through miR‐199a and induces proliferation of adult CMs	26
*Anti‐proliferation microRNAs*							
miR‐1	Mice	‐	β‐MHC‐miR‐1	‐	Embryonic	miR‐1 directly targets **Hand2** to decrease proliferating CMs in the developing heart	47
	Mice	‐	Targeted Deletion of miR‐1‐2	‐	Neonatal Adult	miR‐1 directly targets **Irx5** to regulate the cardiac electrical system	49
miR‐26a	Mice zebrafish	NRVMs	WT mice	injected LNA‐miR‐26a to neonatal mice	Neonatal	miR‐26a inhibits **Ezh2** expression to regulate PRC2‐mediated repression of negative cell cycle regulators and stimulates CMs proliferation	56
miR‐29a	Rats	H9c2 NRVMs	‐	miR‐29a mimics or inhibitors transfection	Neonatal	miR‐29a inhibition leads to neonatal CMs proliferation through targets **CCND2**	57
miR‐29a, miR‐30a and miR‐141	Rats	NRVMs	‐	miR‐29a, miR‐30a and miR‐141 inhibitors transfection	Neonatal	miR‐29a, miR‐30a and miR‐141 negatively regulates CMs through predicted targets **CCNA2** and **CDK6**	59
miR‐34a	Mice Rat	NRVMs	WT mice	miR‐34a mimics and LNA‐based miR‐34a intravenously injection after MI injury	Neonatal Adult	miR‐34a regulates cell cycle activity and death through modulation of its targets including **Bcl2**, **Cyclin D1** and **Sirt1**	60
miR‐99/100 and Let‐7a/c	Mice zebrafish	NMVMs AMVMs	transgenic zebrafish: CMslc2: GFP WT mice	Heart amputation on zebrafish MI injury on mice	Adult	miR‐99/100 and Let‐7a/c inhibits CMs proliferation through down‐regulating **FNTβ/SMARCA5**	62
miR‐128	Mice	NMVMs AMVMs	α‐MHC‐tTA; miR‐128 Tet RE Nkx2.5 Cre; miR‐128 fl/fl α‐MHC‐MCM; miR‐128 fl/fl α‐MHC‐MCM; miR‐128fl/fl; R26R tdTomato	MI injury Neonatal mouse apex resection	Neonatal Adult	miR‐128 inhibition directly up‐regulates expression of **SUZ12**, which decreases p27 expression and activates Cyclin E and CDK2 to promote CMs proliferation	61
miR‐133	Mice	‐	miR‐133a‐1, miR‐133a‐2 dKO mice β‐MHC‐miR‐133a‐2 transgenic embryos	‐	Embryonic Neonatal	Deletion of miR‐133a‐1 and miR‐133a‐2 promotes CMs proliferation through up‐regulating **CCND2**	50
	Zebrafish	‐	transgenic zebrafish: hsp70: miR‐133 hsp70: miR‐133sp	Heart amputation	Adult	miR‐133 inhibits injury‐induced CMs proliferation through targeting ***cx43***	51
miR‐144	Rat	H9c2	‐	‐	‐	miR‐144 inhibits H9c2 cell proliferation through binding to **TBX1** and down‐regulates cell apoptosis through inhibiting the JAK2/STAT1 signalling pathway	94
miR‐195	Mice Rat	H9c2 NRVMs	βMHC‐miR‐195 WT mice	Subcutaneous injection of LNA‐modified miR‐15b and miR‐16	Embryonic neonatal	miR‐195 inhibits CMs proliferation through directly targeting **Chek1**	53, 54

The targets of each miRNA are highlighted with bold values.

Abbreviations: AFVMs: adult feline left ventricular myocytes; AMVMs, adult mice ventricle myocytes; EMCMs, embryonic mouse cardiomyocytes; hiPS‐CMs, human induced pluripotent stem cell‐derived cardiomyocytes; HMCM, adult human primary ventricular cardiomyocytes; I/R, ischaemia reperfusion; LNA, locked nucleic acid; MI, myocardial infarction; NMVMs, neonatal mice ventricle myocytes; NRVMs, neonatal rat ventricle myocytes.

#### 2.1.1 miRNAs promote CMs proliferation

##### miRNAs targeting Hippo‐Yap signal pathway

Microscopy‐based high‐content screening functionally identified that hsa‐miR‐590 and hsa‐miR‐199a effectively increased both DNA synthesis and cytokinesis in neonatal mice and rat CMs.^20^ After myocardial infarction, these miRNAs strongly stimulated cardiac regeneration and significantly recovered cardiac function.^20^ The deep‐sequencing analysis revealed that *Homer1, Hopx and Clic5* are targets of these miRNAs.^20^ Their effects on stimulating CMs proliferation is based on cumulative results on multiple targets. Hippo signal transduction pathway is considered a critical approach to regulate proliferation.^21^ A recent study has shown that a series of miRNAs promote CMs proliferation, including hsa‐miR‐590 and hsa‐miR‐199a, by activating nuclear translocation of YAP and inducing the expression of YAP‐responsive genes.^21^, ^22^ In addition, several miRNAs (including miR‐199a‐3p) also inhibit filamentous actin depolymerization by targeting Cofilin2 and activating YAP nuclear translocation.^22^

The deletion of the miR‐302‐367 cluster using the Cre‐LoxP system expressed during embryonic development confirmed that this cluster is essential for CMs proliferation.^23^ Intracardiac injection of Gel‐miR‐302 stimulates both the wild‐type and Myh6‐MerCreMer:R26R‐Confetti transgenic mice CMs to proliferate.^24^ Furthermore, miR‐302‐367 cluster, hsa‐miR‐590 and hsa‐miR‐199a, exert their pro‐proliferative effects on CMs by targeting components (Lats2, Mob1 and Mst1) of the Hippo signalling pathway.^23^

##### miRNAs regulate the cell cycle to promote CMs proliferation

The regulation of cyclin, cyclin‐dependent kinase and regulators highly expressed in the foetal stage, significantly stimulate adult CMs to re‐enter cell cycle.^25^ The majority of these proteins are targets of miRNAs**.** miR‐1825 is one of the miRNAs screened by the above approach^20^ and a master regulator of miR‐199a.^26^ Transfecting with miR‐1825 mimics markedly increases the proliferation of adult mice CMs.^26^ MiR‐1825 has been reported to reduce the mitochondrial numbers and destroy their function by direct inhibition of NDUFA10 and cell cycle genes.^26^

miR‐204 stimulates human CM progenitor cells to proliferate and differentiate.^27^ Transgenic mice with highly cardiac expression of miR‐204 exhibit a thicker ventricular wall, which is associated with CMs proliferation rather than hypertrophy during heart development by directly targeting Jarid2,^28^ while Jarid2 binds to the promoter of cyclin D1 and represses its expression.^29^

MiR‐210, up‐regulated in many cardiac diseases, exerts its beneficial effects against ischaemic injury when injected into the myocardium.^30^ Transfection adult rat CMs with miR‐210 significantly increases the amount of CMs and inhibits apoptosis as well.^31^ Overexpression of miR‐210 in transgenic mice results in recovery against injury and also promotes CMs proliferation and angiogenesis.^31^ In silico analysis indicates that APC (adenomatous polyposis coli)‐cell cycle inhibitor is involved in the canonical Wnt signalling pathway, which is a target of miR‐210.^31^

miR‐294 is highly expressed during embryonic cardiac development and rapidly declines after maturation, which has been found to promote both neonatal rat ventricular myocytes (NRVMs) and feline adult CMs to enter the cell cycle.^32^ In another study, miR‐294 mechanically blunted the Wee1, a negative regulator of the cell cycle,^25^ to increase the activity of the cyclin B1/CDK1 complex and improve CMs proliferation.^32^

MEF2A‐regulated Gtl2‐Dio3 non‐coding RNAs plays an important role in CMs differentiation and maturation.^33^ miR‐410 and miR‐495, the subsets of Gtl2‐Dio3 miRNA mega‐cluster, promote NRVMs to proliferate by directly inhibiting CITED2.^33^ CITED2 is a transcriptional coactivator that promotes the expression of the cell cycle inhibitor *Cdkn1c/p57/Kip2*.^33^

miR‐499, a cardiac abundant miRNA, promotes mouse P19CL6 cells to differentiate to CMs and inhibits apoptosis in NRVMs during late stage of differentiation.^34^ miR‐499 functions through direct targeting of 3’ UTR of SOX6, which negatively regulates the transcription of the cyclin D1^34^ and plays a critical role in CMs development.^35^

##### Exercise‐induced miRNAs regulate CMs proliferation

Exercise induces physiological cardiac growth evidenced by increased proliferation markers and protection of the heart against pathological remodelling. miR‐17‐3p is induced by exercise and protects the heart against ventricular remodelling.^36^ Inhibition of miR‐17‐3p can attenuate CMs hypertrophy and inhibit their proliferation.^36^ Besides, miR‐17‐3p directly targets tissue inhibitor of metalloproteinase‐3 (TIMP3) to induce CMs proliferation via EGFR/JNK/SP‐1 signalling^37^ and indirectly regulates PTEN to promote CMs hypertrophy.^36^ The expression level of miR‐222 is also up‐regulated in exercise models.^38^ Overexpression of miR‐222 is sufficient to induce neonatal CMs physiological growth, cellular hypertrophy and proliferation by reducing the expression of p27, negatively regulating the cell cycle and transcription factor HIPK1^39^ and inhibiting apoptosis.^38^ Cardiac‐specific expression of miR‐222 protects against cardiac remodelling and dysfunction after ischaemic injury.^38^ The negative function of miR‐222 was further demonstrated by multi‐isotope imaging mass spectrometry (MIMS) to identify newly formed CMs in the exercise model, which could be completely blocked by inhibition of miR‐222.^40^

##### Other mechanisms

miR‐17‐92 cluster, known as OncomiR‐1, is required for CMs proliferation in the embryonic and postnatal mouse hearts.^41^ Overexpression of miR‐17‐92 induces CMs proliferation in embryonic, postnatal and adult heart and protects the adult heart from myocardial infarction through targeting *Pten*.^41^ MiR‐19a/19b, family members of miR‐17‐92 cluster, are highly expressed in heart failure patients.^42^ Overexpression of miR‐19a/19b promotes CMs proliferation, reduces apoptosis and blocks inflammation through targeting *Pten, Bim1* and *SOCX1/3*. MiR‐19a/19b protect the adult heart in two distinctive phases after myocardial infarction: early‐phase and long‐term protection.^42^ Furthermore, miR‐25 also belongs to an oncogene named MCM7, it promotes CMs growth and migration by targeting Bim.^43^

miR‐31‐5p is up‐regulated in P10 CMs compared to P0, but it promotes NRCMs proliferation through targeting RhoBTB1,^44^ a subfamily of the Rho small GTPases.^45^ This up‐regulation of miR‐31‐5p is probably a compensatory mechanism of the CMs in response to exiting the cell cycle.

Unbiased miRNA‐sequencing indicated that miR‐486 was enriched in striated muscle and was up‐regulated in neonatal patients with hypoplastic left heart syndrome which was confirmed by sheep dilated right ventricle.^46^ The ventricle of neonatal mice treated with miR‐486 mimics exhibited increased growth of the ventricles without changes in wall thickness and CMs proliferation.^46^ Previously, iTRAQ‐based mass spectrometry proteomics studies indicated that Stat1 was one of the most up‐regulated proteins after miR‐486 mimic treatment.^46^ miR‐486 indirectly decreased FoxO1 and Smad signalling and increased the Stat1 expression level associated with Gata4 and Serum Response Factor (Srf) to stimulate CMs proliferation.^46^

#### 2.1.2 miRNAs inhibit CMs proliferation

##### The role of miR‐1‐2/miR‐133a‐1 and miR‐1‐1/miR‐133a‐2

The cardiac‐ and skeletal muscle‐specific miRNA genes miR‐1‐1 and miR‐1‐2 are specifically expressed in ventricle during cardiogenesis and activated during differentiation.^47^ Transgenic mice with β‐myosin heavy chain (MHC) promoter highly express miR‐1 at E9.0 resulting in the thinner ventricular wall and less CMs proliferation via targeting Hand2,^47^ which is required for the expansion of the embryonic cardiac ventricles.^48^

Targeted deletion of miR‐1‐2 without affecting the resident gene Mib1 showed ventricular septal defect at E15.5 and some (~15%) mice survived to 2‐3 months would suddenly die due to electrophysiologic defects as a consequence of direct inhibition of Irx5 by miR‐1‐2.^49^ Besides, most adult miR‐1‐2 mutants have a thicker ventricular wall due to the increased proliferation of CMs. The effect on ventricular wall is consistent with overexpressed miR‐1 in the heart.^49^

miR‐133a‐1 and miR‐133a‐2 have identical sequences and are highly expressed in the heart.^50^ The two miRNAs are transcribed as bicistronic transcripts with miR‐1‐2 and miR‐1‐1, respectively, in skeletal and cardiac muscle.^50^ Mice deleting single gene were normal, whereas double knockout mice died during late embryonic or neonatal stage due to ventricular septal defect, along with enhancement of CMs proliferation, apoptosis and aberrant expression of smooth muscle genes in the heart.^50^ Cyclin‐D2, a positive regulator of cell cycle, is a target of miR‐133a‐1 and miR‐133a‐2.^50^ Microarray data indicated that miR‐133 was reduced during regeneration after resection of the ventricular apex in zebrafish.^51^ Transgenic overexpression of miR‐133 by heating shock single time after resection in short‐term or daily in the long‐term inhibited cardiac regeneration due to decreased CMs proliferation.^51^ However, the deletion of miR‐133 significantly increased the proliferation index of CMs and restored the myocardium through persistent inhibition of miR‐133.^51^ Besides the regulators of the cell cycle, pharmacological inhibition and EGFP sensor interaction studies indicated that *cx43*, a component of the cell junction, was a miR‐133 target.^51^ Another study on sheep showed miR‐133 expression in heart enhanced while it's direct target gene IGF1R's expression decreased with age.^52^ The expression profile of other targets of miR‐133 such as *CCND2, SRF, PGAM1* and *GJA1(Cx43)* did not show the reverse tendency of miR‐133; hence, the regulatory effect of miR‐133 could be through an indirect signalling pathway.^52^

##### Cell cycle regulators

miR‐195 is one of the miR‐15 families with 6 miRNAs sharing a similar seed region and is the most up‐regulated miRNA in P1 and P10 mice.^53^ Transgenic mice overexpressed miR‐195 during embryonic would partly die on the consequences of the large apical ventricular septal defect and ventricular hypoplasia.^53^ Though the survival parts had a normal cardiac function, they showed fewer proliferating CMs and depressed cardiac function.^54^ However, neonatal mice receiving LNA (locked nucleic acid)‐modified miR‐15b and miR‐16 showed more CMs mitosis re‐entry and progression without cytokinesis.^53^ Furthermore, LNA‐modified miRNA injection from neonatal to adult improved heart function and stimulated CMs proliferation after MI injury.^54^ RISC‐seq confirmed that the miR‐15 family negatively regulated cell cycle by directly targeting *Chek1* (checkpoint kinase 1), a conserved target between humans and mice, and was required for G_2_/M DNA damage checkpoint.^53^, ^55^

Global gene profiling of injured mouse and zebrafish hearts has revealed that miR‐26a is down‐regulated in the injured zebrafish hearts while keeping constant in the injured mouse hearts.^56^ LNA‐mediated inhibition of miR‐26a promotes neonatal CMs proliferation by targeting Ezh2, a component of polycomb repressive complex 2 (PRC2), which exerts suppressive functions on negative regulators of the cell cycle.^56^

miR‐29a was found to be highly up‐regulated in postnatal‐4‐week rat CMs compared to neonatal. Inhibitors of miR‐29a could stimulate H9c2 and NRVMs to proliferate through targeting CCND2, a positive regulator of cell cycle.^57^ In addition, CCND2 was also a target of an anti‐proliferating miRNA let‐7i‐5p.^58^ Another study found that miR‐29a was up‐regulated in purified adult rats CMs compared with neonatal and postnatal as well as miR‐30a and miR‐141 families.^59^ Anti‐miR of miR‐29a, miR‐30a and miR‐141 enhanced the cell cycle re‐entry of NRVMs and the predicted targets were Ccna2 and CDK6.^59^

miR‐34a is a regulator of age‐associated physiology and prevents heart from regenerating in MI injury and its negative effect on cell proliferation is by direct targeting Bcl2, Cyclin D1 and Sirt1.^60^

RNA‐sequencing analysis of P1, P7 and P28 mice cardiac ventricles indicated that miR‐128 expression was increased upon growth.^61^ Cardiac‐specific overexpression of miR‐128 in early‐stage led to the enlargement of the ventricle, reduced function and decreased regeneration after apex resection due to decreased proliferating of CMs.^61^ Conditional deletion of miR‐128 in neonatal mice showed similar heart size but smaller and more proliferating CMs.^61^ Knockout of miR‐128 in adult mouse hearts resulted in more proliferating and differential CMs under basal conditions as well as improved heart regeneration at injury conditions.^61^ miR‐128 down‐regulated the expression of chromatin modifier SUZ12, which decreased p27 (cyclin‐dependent kinase inhibitor) expression and activates the positive cell cycle regulators Cyclin E and CDK2 to promote CM proliferation.^61^

##### Other mechanisms

miR‐99/100 and let‐7a/c expression was markedly down‐regulated during regeneration in zebrafish while their targets *fntb* and *smarca5,* were found to be conserved in the mammalian genome.^62^ Intracardiac injection of miR‐99/100 mimics reduced BrdU incorporation after heart amputation and inhibition of miR‐99/100 led to cardiac hypertrophy in adult zebrafish.^62^ Experimental down‐regulation of miR‐99/100 and Let‐7a/c or up‐regulation by FNTβ/SMARCA5 would lead mammalian CMs to a differentiation state and then proliferate.^62^

### 2.2 LncRNAs regulate CMs proliferation

**FIGURE 3:**
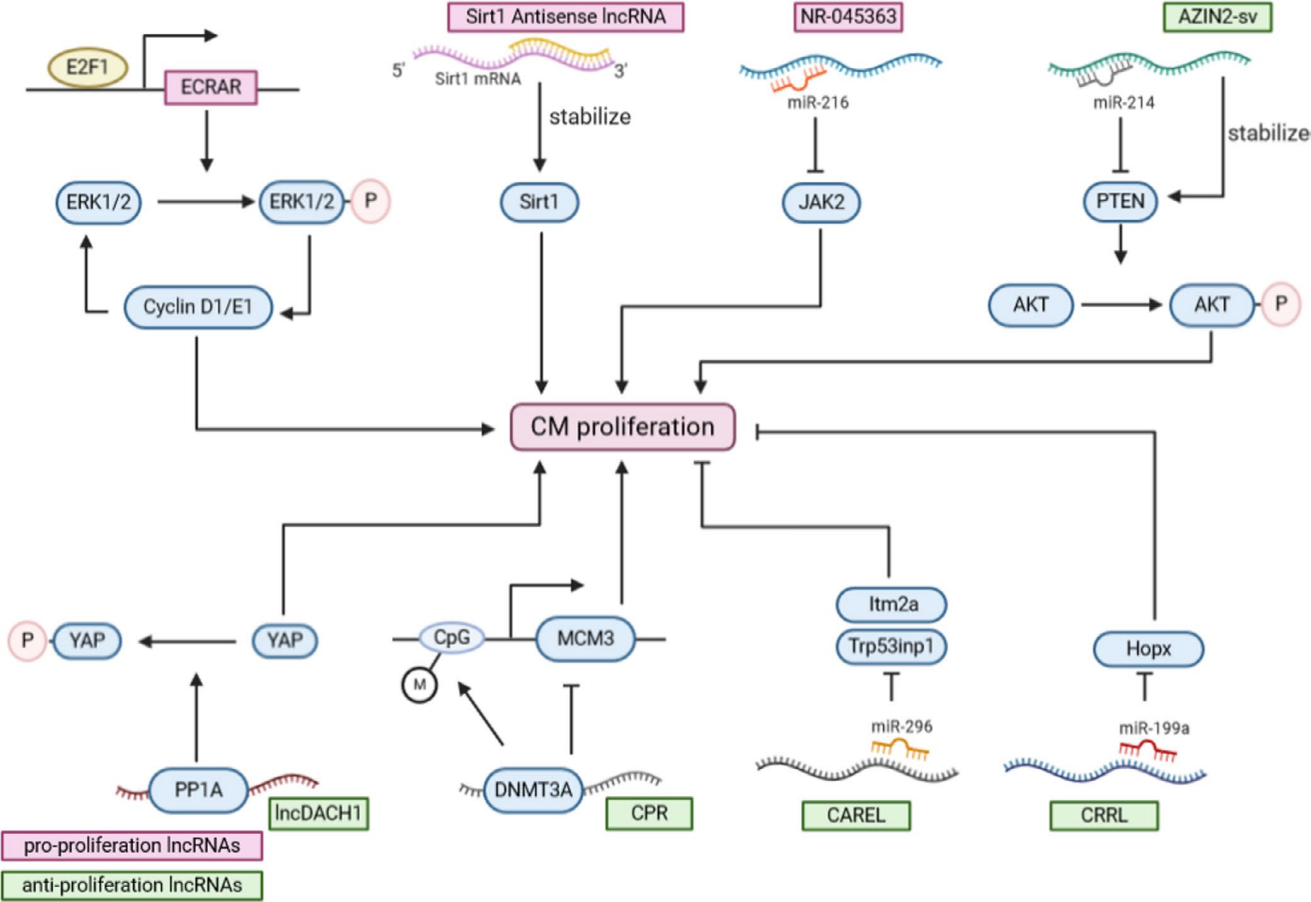
The mechanisms of lncRNAs in CM proliferation. The pink boxes indicate the lncRNAs that promotes the proliferation of CM, and the green boxes indicate the lncRNAs that inhibits proliferation. The round boxes represent the protein targets they regulate. The specific mechanisms are shown in Table 2. (Created with BioRender.com)

More recently, lncRNAs have come into the spotlight due to their roles in regulating gene expression and biological processes. LncRNAs are generated by RNA polymerase Ⅱ, 5’‐capped, spliced and 3’‐ polyadenylated (except some specific non‐polyadenylated lncRNAs^63^, ^64^)‐but they are not translated into proteins and are expressed at a relatively low level.^65^ Based on their gene location, they are divided into six subgroups: sense, sense intronic, antisense, bidirectional, enhancer or intergenic lncRNAs.^65^ However, this standard of classification is not enough due to the abundant amount of lncRNAs.^10^ Therefore, based on their functions, nuclear‐expressed lncRNAs are divided into signal, decoy, guide or scaffold and enhancer lncRNAs.^66^ The rest of the cytosol‐expressed lncRNAs support the following functions: they can be integrated into a complex of ribonucleoprotein (RNP) and trafficking, act as a sponge to sequester miRNAs and combined with mRNAs to stabilizing or destabilizing them.^66^ The specific definition of this classification has been reviewed in detail previously.^65^, ^66^ Here, we discuss the role of lncRNAs in regulating CMs proliferation and cardiac repair as well as their potential therapeutic role. The specific regulating mechanism of each lncRNA is depicted in Figure 3.

#### 2.2.1 LncRNAs promote CMs proliferation

Transcriptome analysis revealed that ECRAR was significantly up‐regulated and was more active in the chromatin state in the foetal human heart compared with adults.^67^ Adenovirus‐ or AAV‐mediated overexpression of ECRAR was found to promote postnatal and adult CMs to re‐enter cell cycle without leading to hypertrophy at baseline or subjected MI injury.^67^ Besides, ECRAR knockdown in neonatal rat using shRNA showed decreased proliferation of CMs which was further confirmed in human AC16 cells.^67^ E2F1 activates ECRAR transcription which promotes the phosphorylation of ERK1/2, an important regulator of G1/S transition.^67^ Phosphorylated ERK1/2 stimulates the expression of cyclin D1, cyclin E1 and E2F1 to form positive feedback.^67^

Sirt‐1 antisense lncRNA is highly expressed in the embryonic stage and rapidly decreases after birth.^68^ Adenovirus‐mediated gain‐of‐function promotes NRVMs to undergo mitosis, karyokinesis and sarcomere disassembly.^68^ LNA‐mediated loss‐of‐function approach in neonatal and AAV9‐mediated overexpression of this lncRNA in adult mice, both indicated that this lncRNA was required and sufficient to induce CMs proliferation.^68^ Elevated expression of this lncRNA also enhances survival rate, improves cardiac function, reduces infarct area and inhibits fibrosis after MI.^68^ Complementary with 3'UTR of Sirt1 mRNA, Sirt1 antisense lncRNA interacts with Sirt1 mRNA and augments its stability and pro‐proliferating ability.^68^

NR‐045363 is an antisense lncRNA to human CDK6, which is reported to be down‐regulated in the embryo but up‐regulated in adults 7 days after apex resection and correlates with CMs proliferation.^69^ AAV‐mediated overexpression improves cardiac function and reduces scar size due to increased CMs proliferation.^69^ miR‐216 is sequestered by NR‐045363 and proven to inhibit CMs proliferation by inactivating the JAK/STAT3 signal pathway.^69^

Cardiovascular diseases are another factor that changes the proliferation status of CMs.^70^ In chronic heart failure (CHF) patients, the expression level of LUCAT1 is decreased markedly.^70^ LUCAT1 has been demonstrated to sponge miR‐612 which targets HOXA13 in AC16 cell lines to stimulate CMs proliferation.^70^

#### 2.2.2 LncRNAs inhibits CMs proliferation

Contrary to LUCAT1, LncDACH1 is up‐regulated in CHF patients and postnatal hearts. Cardiac‐specific overexpression of LncDACH1 impedes cardiac repair after apical resection while loss‐of‐function in the heart reactivates CMs entry into the cell cycle.^71^ LncDACH1 is directly bound to the protein phosphatase 1 catalytic alpha (PP1A) subunit to limit its activity in dephosphorylation while enhancing YAP1 phosphorylation and reducing its translocation into the nucleus to inhibit CMs proliferation.^71^

Nuclear abundant CPR is highly expressed in adult ventricular CMs compared to embryonic mice heart.^72^ CPR global knockout mice showed normal morphology and heart function but increased CMs proliferation in both postnatal and adult mice under physiological status.^72^ Furthermore, the deletion of CPR promoted adult heart regeneration after MI injury.^72^ Cardiac‐specific overexpression of CPR led to the hypertrophic phenotype under physiological condition, increased scar and poor cardiac function after MI injury, which was further confirmed in the AAV9 overexpression system.^72^ CPR acted as a guide to recruit DNMT3a to methylate the CpG island which inhibited the expression of MCM3.^72^

Microarray analysis has shown that CAREL is up‐regulated with growth and development.^73^ Transgenic mice with cardiac‐specific overexpression of CAREL impede CMs proliferation and cardiac regeneration after apex resection which has been confirmed in the intracardiac injection of CAREL adenovirus.^73^ However, CAREL deletion mediated by adenovirus reduces the scar size, improves cardiac function and enhances CMs cell cycle re‐entry after MI injury.^73^ CAREL, expressed in the cytoplasm of CMs, acts as an endogenous competitor of miR‐296 and promotes CMs proliferation by directly targeting Trp53inp1 or Itm2a.^73^

RNA‐seq data from human foetal and adult heart revealed that the expression of AZIN2‐sv and CRRL were correlated with cell cycle‐related protein‐coding genes and increased with age.^74^, ^75^ Adenovirus‐mediated gene regulation of these lncRNA revealed that they are negative regulators of CMs proliferation.^74^, ^75^ Loss‐of‐function assays in adenovirus and AAV9 neonatal and adult rats, respectively, revealed stimulated cardiac regeneration through restraining ventricular remodelling, improving heart function and activating CMs proliferation against MI injury.^74^, ^75^ Both lncRNAs are highly expressed in the cytoplasm and act as competing endogenous RNAs to inhibit proliferation by sponging miRNAs. AZIN2‐sv sponged miR‐214 to release phosphatase and tensin homolog (PTEN) which blocked the activation of PI3K kinase/Akt pathway, inhibited CMs proliferation and enhanced the stability of PTEN.^74^ CRRL sponged miR‐199a by targeting Hopx, a negative regulator of the cell cycle.^75^

### 2.3 CircRNAs regulate CMs proliferation

CircRNAs (circular RNAs) are circularized by connecting the 3’ end to 5’ end to provide stability compared with non‐circular RNAs and thus play an important role in the regulatory pathway.^76^, ^77^ Superenhancer associated circRNA circNfix is highly expressed in adult hearts and also highly expressed in the cytoplasm of CMs.^78^ SiRNA‐mediated knockdown and adenovirus‐mediated overexpression revealed that circNfix was a negative regulator of CMs proliferation.^78^ The knockdown of circNfix by AAV9 packaging shRNA significantly facilitates adult CMs proliferation and dedifferentiation marked by increased RUNX1.^78^ CircNfix exerted its anti‐regenerating effect through two independent pathways– inhibiting CMs proliferation and angiogenesis.^78^ First, transcription factor Meis1 binds to the superenhancer of CircNfix and promotes transcription and cyclizing of Nfix.^78^ CircNfix then combines with Ybx1‐a positive regulator of CyclinA2/B1‐and Nedd4l, to the consequence of ubiquitination and degradation of Ybx1.^78^ Second, circNfix acts as a sponge to absorb miR‐214 which directly targets Gsk3β and promotes the expression of β‐catenin to inhibit angiogenesis.^78^

Highly expressed in foetal and neonatal heart, CircHipk3 can facilitate cardiomyogenesis and angiogenesis.^79^ CircHipk3 could increase the stability of Notch1 intracellular domain (N1CID) by acetylation and prevent its degradation to stimulate CMs proliferation.^79^ CircHipk3 also acts as a sponge for miR‐133a to increase the expression level of connective tissue growth factor (CTGF), then activates endothelial cells.^79^ The summary of LncRNA and CircRNA in CMs proliferation are listed in Table 2.

**TABLE 2:** Summary of the effect of lncRNAs and circRNAs on cardiomyocytes proliferation

LncRNAs	In Vitro		In Vivo				Mechanism	Ref
	Cells	Transfection	Animals	Operation	Administration	Stage		
*Pro‐proliferation lncRNAs*								
ECRAR	NRVMs AC16 cells AMVMs	Adenovirus shRNA	WT Rats	MI injury	Adenovirus AAV9	postnatal adult	ECRAR promotes CMs proliferation through a positive feedback that being up‐regulated by E2F1 and promoting phosphorylation of ERK1/2 which resulting cyclin D1, cyclin E1 and E2F1 activation	67
Sirt1 antisense lncRNA	NMVMs	Adenovirus LNA	WT mice	MI injury	AAV9 LNA	postnatal adult	Sirt1 antisense lncRNA interacts with the 3' UTR of Sirt1 mRNA and enhances its stability to promote CMs proliferation	68
NR_045363	embryonic CMs NMVMs	lncRNA smart silencer Adenovirus	WT mice	AR	AAV9	neonatal	NR_045363 plays positive rule on cardiac regeneration by directly binding to miR‐216a and activating the miR‐216a/JAK2‐STAT3 pathway	69
*Anti‐proliferation lncRNAs*								
LncDACH1	‐	‐	LncDACH1 TG LncDACH1 cKO	AR MI injury	Adenovirus	juvenile adult	LncDACH1 directly bound to PP1A to limit its activity of dephosphorylation meanwhile enhanced YAP1 phosphorylation and reduced its translocation into nuclear to inhibit CMs proliferation	71
CPR	NMVMs hESC‐derived CMs AMVMs	siRNA	CPR KO α‐MHC‐CPR Tg WT mice	MI injury	AAV9	postnatal adult	CPR directly interacting and recruiting DNMT3A to MCM3 promoter CpG sites to inhibit CMs proliferation	72
CAREL	NMVMs hESC‐derived CMs	Adenovirus	Myh6‐CAREL Tg WT	MI injury	Adenovirus miRNA mimics miRNA inhibitors	postnatal adult	CAREL acts as an endogenous competitor of miR‐296, which promotes CMs proliferation via directly targeting Trp53inp1 or Itm2a	73
CRRL	NRVMs	Adenovirus	WT Rats	MI injury	Adenovirus AAV9	postnatal adult	CRRL acts as a ceRNA by directly binding to miR‐199a‐3p and thus increasing the expression of Hopx to negatively regulate CMs proliferation	75
AZIN2‐sv	NRVMs AC16 cells	Adenovirus	WT Rats	MI injury	Adenovirus	postnatal adult	AZIN2‐sv directly binds to PTEN to increase its stability and acts as a micro‐214 sponge to release PTEN which block activation of the PI3K/Akt pathway to inhibit CMs proliferation	74
*CircRNAs*								
CircNfix	NMVMs HL‐1 KD AMVMs	siRNA	WT mice CRISPR‐Cas9 knock in mice	MI injury	AAV9 Adenovirus	postnatal adult	circNfix is transcripted by Meis1' binding to the superenhancer and then promote Ybx1 ubiquitination and degradation to repress expression of cyclin A2/B1 to inhibit CM proliferation. Deletion of circNfix could increase the expression of miR‐214, which directly targeting Gsk3β to promote angiogenesis to facilitate cardiac regeneration	78
CircHipk3	NMVMs AMVMs	siRNA Adenovirus	WT mice	MI injury	AAV9	postnatal adult	CircHipk3 could increase the stability of N1CID by acetylation and prevent its degradation to stimulate CMs proliferation. Meanwhile, CircHipk3 also acts as a sponge for miR‐133a to increase the expression level of CTGF, then activates endothelial cells	79

Abbreviation: AR, apex resection.

## 3 RNA‐BASED THERAPEUTIC STRATEGIES

Given the significance of non‐coding RNA in regulation of CMs proliferation, the therapeutic potential of non‐coding RNAs has aroused extensive research interests. The first question is how to direct the non‐coding RNAs into the specific tissue or cell to play their biological roles? During the last two decades, viral particles were found to be effective tools to package plasmid containing therapeutic non‐coding RNAs. Besides, many oligonucleotides were also designed and modified to enhance their affinity and stability and strengthen the curative effect.

### 3.1 Viral vector‐based gene therapy

#### 3.1.1 Adenoviral‐based gene delivery

Adenoviruses are double‐stranded DNA viruses that packaged in a high‐affinity protein capsid. Due to their high transfection efficiency and robust transgene expression, they are extensively used in scientific research. However, the characteristic of transient expression limits their application in the treatment of diseases. Adenovirus vectors containing shAZIN2‐sv injected into the myocardium has been reported to preserve adult rats’ cardiac function, reduce infarct area and promote angiogenesis from 14 to 60 days after MI injury.^74^ Besides, adenoviral proteins elicit hosts’ immune response and this is one of the major hurdles limiting its therapeutic application.

#### 3.1.2 Adeno‐associated virus‐based gene delivery

Adeno‐associated viruses have a single strand DNA genome that does not integrate into the host genome. Currently, more than 10 serotypes are known and used in gene therapy. Another AAV’s feature is the serotypes with different organotropism. AAV serotype 9 showed the best transgene expression and distribution when systemically delivering different AAV serotype to mice.^80^ AAV9 has been widely used in rodents while AAV6 has been used to cure cardiac injury in pigs.^81^ Though AAV9‐packaged transgenes are highly expressed in cardiac, other organs such as the liver and lungs have comparable expression due to systemically delivery. As a consequence, AAV9 is delivered through intracardiac injection.^20^, ^26^, ^42^, ^62^, ^68^, ^75^, ^81^ 1‐3×10^11^ of AAV particles by a single dose were employed in these articles.

The episomal circular form of recombinant AAV results in long time expression ranging from days to months. AAV 9‐mediated cardiac‐targeted delivery of miR‐19a/19b soon after MI injury provides long‐term protection lasting from 7 days to 2‐3 months after injection.^42^ Intracardiac injection AAV9‐anti‐miR‐99/100 or AAV9‐anti‐Let‐7a/c in the border of the infarcted area provides prolonged cardiac protection for up to 90 days after MI injury.^62^

Non‐coding RNAs that inhibit CMs proliferation can also be used as treatment through their shRNA packaged by AAV particles. Injection of AAV9‐shcircNfix into the peri‐infarct area of adult mice resulted in significant improvement in the ejection fraction post‐MI.^78^

In a previous study, adult mice subjected to MI surgery were injected with AAV9 vectors expressing hsa‐miR‐590 and hsa‐miR‐199a into peri‐infarcted area. This resulted in improved cardiac function from 12 days to 1‐2 months after injection.^20^ However, in mammals, this long‐term expression of transgene may be cause detrimental effects. Delivery of hsa‐miR‐199a through an AAV serotype 6 vector 1‐month post‐MI, treated pigs showed marked improvements in both global and regional contractility, increased muscle mass and reduced scar size.^81^ It is worth noting that in pigs treated with the miR‐199a, although 30% showed a continuous improvement in cardiac morphology and function with a recovery period lasting for two months, 70% sudden died 7‐8 weeks after MI.^81^ The electrocardiogram results revealed that the deaths were caused by accelerated heart rate which led to ventricular fibrillation.^81^ However, immune response elicited by AAV injection may in human also hindering the clinical use.^82^

#### 3.1.3 Lentiviral‐based gene delivery

Unlike adenovirus or AAV which possess DNA genome, lentivirus is a kind of complex retrovirus. Compared to simple retroviruses, lentivirus genomes contain regulatory genes, auxillary genes and nuclear location signal which help the lentivirus to enter the nuclear pore and to integrate into host's genome.^83^ Hence, lentivirus can be used to transfect cells that do not undergo mitosis in vitro. However, expression of transgenes packaged in lentiviruses does not last long in vivo. Single intracardiac injection of lentiviruses packaging anti‐miR‐99/100 and anti‐Let‐7a/c into peri‐infarcted region significantly improved cardiac function 14 days after MI but this beneficial effect only lasted for short time.^62^

### 3.2 Oligonucleotides‐based gene therapy

Although virus vectors are a powerful tool for expressing non‐coding RNAs, several issues such as the short duration of expression, risk of infecting other unintended organs and possibility of triggering an immune response limit their clinical application. Because the dedifferentiation of CMs is a prerequisite step determining the ability of CMs to repair cardiac injuries, prolonged expression of pro‐proliferating miRNAs may result in adverse effects.

#### 3.2.1 MiRNA mimics and lipid formulation

Synthetic oligonucleotides might be a more promising alternative. This is because they produce prolonged proliferative effect without long‐term potential adverse effects.^84^ Up‐regulation of some miRNAs that improve CMs proliferation using miRNA mimics maybe an alternative approach to treat cardiovascular disease. MiRNA mimics are double‐strand, chemically modified oligonucleotides that do not undergo natural miRNA biogenesis but have the same biology function such as inhibiting the expression of target genes.^85^, ^86^ Furthermore, 2’ ‐O‐methoxyethy of ribose, 5’ Cholesterol and phosphorothioates backbone modified miRNA agonist called agomir was found to significantly improve the nuclease resistance and affinity of mimics. Mice injected with miR‐17‐3p agomir through tail vein were protected from adverse remodelling after cardiac I/R injury.^36^ Oligonucleotides are much smaller than biomacromolecules such as proteins or ribonucleic acids, but they are bigger than some small molecules (<500 Da) which can passively diffuse across cellular membranes. Mimics or agomirs are larger than 14kDa and have numerous charges. Therefore, to enhance their cellular uptake, they should be packaged into some nanoparticles. Five kinds of lipid formulations were used to deliver pro‐proliferation miR‐199a‐3p mimics. RNAiMAX was found to be the most effective (transfection efficiency > 80%) and less toxic formulation.^84^ The expression level of miRNA significantly increased 3 days after intracardiac injections and the inhibition effect on their targets could maintain for 8‐12 days.^84^

Overexpression of miR302‐367 cluster in adult mice heart reduced the scar formation following MI injury but it did not improve the heart function.^23^ This phenomenon may be caused by the persistent expression of pro‐proliferation miRNAs resulting in dedifferentiation of many CMs. Notably, transient expression of miR302b/c achieved by tail‐vein injection of mimics and RNALancerII neutral lipid formulation for 7 consecutive days markedly reduced scar formation, stimulated CMs proliferation, improved angiogenesis and heart function 50 days after MI.^23^

Intravenous tail‐vein injection of miR‐19a/19b mimics, embedded in neutral lipid emulsion RNALancerII and RNAiMax, induced CMs proliferation and stimulated cardiac regeneration following MI.^42^ miRNA mimics can enter CMs 12 hours after injection and the expression level of miR‐19a/b was detected 4 days after injection but were undetectable at 1 month later.^42^

Lipid nanoparticle delivery of miR‐708 using RNALancerII injected via tail vein protected against cardiac injury induced by isoproterenol.^87^ The expression level of miR‐708 was up‐regulated for 16 days which is sufficient to inhibit hypertrophy and reduce fibrosis for 5‐10 days following ISO treatment.^87^

#### 3.2.2 Locked nucleic acid

Locked nucleic acid (LNA) means the ribose sugar is locked in a C3’‐endo conformation by the introduction of a 2’‐O‐, 4’‐C‐methylene bridge to form 2’ sugar modification.^88^ This modification improved the affinity of complementary RNA. A previous study injected LNA‐miR‐294‐3p mimic formulated with RNALancerII into the heart once soon after MI injury.^32^ They found that expression level of miR‐294‐3p significantly up‐regulated two days after injection. This was accompanied by increased CMs proliferation but the protective effect only lasted for 2‐3 weeks as the infarct size was not reduced 8 weeks after MI injury.^32^

LNA can also be used to inhibit miRNAs. This is achieved by complementary base pairing to form a DNA‐RNA hybrid that activates RNase H‐dependent degradation of target RNA.^89^ Injection of LNA‐based anti‐miR‐34a through tail vain down‐regulated the expression level of miR‐34a for more than 7 days and improved adult heart function, delayed remodelling and reduced the formation of fibrosis scars 7 days after MI injury.^60^

#### 3.2.3 Hydrogel‐based delivery

Another approach to deliver cholesterol‐modified miR‐302 using shear‐thinning, injectable hydrogels based on the guest‐host interaction of modified hyaluronic acid (HA) by intracardiac injection after MI could improve heart function for 4 weeks.^24^

A biocompatible injectable gel composed of gelatin and silicate was used to deliver viral particles.^26^ The gel prevented the viral particles from being rapidly metabolized by the beating heart and allowed the slow release of the particles from the gel to enhance therapeutic effects.^26^ Simultaneous injection of gel and AAV‐miR‐1825 resulted in significant reduction in scar size, promoted peri‐infarct region adult CMs proliferation and improved overall cardiac function up to 28 days after MI.^26^ The detail of miRNAs therapy is listed in Table 3.

**TABLE 3:** Therapeutic role of microRNAs in cardiac regeneration

MicroRNAs	Animal model	Therapeutic methods		Dosage	Delivery method	Pharmacokinetics	Cardiac function improvement time window	Tissue specificity	Ref
miR‐199a miR‐590a	Mice with MI injury	AAV9‐Hsa‐miR‐590a AAV9‐Hsa‐miR‐199a		1 × 10^11^ viral genome particles per animal	Intracardiac injection	‐	12‐60 d	‐	20
	Mice with MI injury	RNA mimics	Lipofectamine RNAiMAX	20 μL mix of miRNA and lipids (ratio 1:1 in volume)	Intracardiac injection	2‐20 d	1‐8 weeks	heart liver kidney	84
	Pigs with MI injury	AAV6‐Hsa‐miR‐199a‐3p and Hsa‐miR‐199a‐5p pri‐miRNA		2 × 10^13^ viral genome particles per animal	Intracardiac injection	28 d	4 weeks	heart	81
miR‐19a/19b	Mice with MI injury	RNA mimics	Max Suppressor in vivo RNALancerII	10 μg mimic in 50 μL mixture/heart, each injection site is about 15 μL	Ventricle muscular wall injection, three sites around the infarcted area	12 h‐1 mo 4 d highest	2‐4 weeks 4 mo	‐	42
				10 μg mimic in 100 μL mixture per mouse	Single dose per day intravenous tail‐vein injection for 3 d, beginning 6 h after MI		2‐14 weeks	heart	
			Lipofectamine RNAiMax	100 μL mixture of miRNA and RNAiMax (ratio 1:1 in volume)	Single dose per day intravenous tail‐vein injection for 3 d, beginning 6 h after MI	‐	2‐14 weeks	‐	
		AAV9‐miR‐19a/19b		2 × 10^11^ viral genome particles per heart in total volume of 50 μL	Ventricle muscular wall injection, three sites around the infarcted area	72 h‐3 mo 7 d highest	7 d‐3 mo	‐	
miR‐17‐3p	Mice with I/R injury	agomir	‐	10 mg/kg, every 3 d for 4 weeks starting 24 h after reperfusion	Tail‐vein injection	‐	‐	‐	36
miR‐302/367	Mice with MI injury	miR302b/c RNA mimics	Max Suppressor in vivo RNALancerII	a single dose of 10‐mg NLE‐formulated miRNA mimics starting 1 day after MI and continuing daily for 7 d	Intravenous tail‐vein injection	8‐24 h	50 d	heart lung	23
		cholesterol‐modified miR‐302	guest–host HA hydrogel system	2 × 5 μL	Intracardiac injection, inferior and lateral to the peri‐infarcted region	within 28 d	4 weeks	lung	24
miR‐294	Mice with MI injury	LNA‐miR‐294 mimics	Max Suppressor in vivo RNALancerII	1 mg/kg	Single location intramyocardial injection immediately after MI	increased 2 d after injection	2‐3 weeks	‐	32
		doxycycline‐inducible AAV9‐miR‐294 vector		1.08 × 10^12^ viral particles	Intracardiac injection at 4 different locations of peri‐infarcted region	7 d	8 weeks	lung liver	
miR‐708	Mice with cardiac injury induced by ISO	miR‐708 mimics	Max Suppressor in vivo RNALancerII	0.4 mg/kg, RNA mimic was administered daily for 6 d with ISO treatment	Tail‐vein injection	3‐16 d	5‐15 d	heart kidney lung	87
miR‐1825	Mice with MI injury	AAV‐miR‐1825		1 × 10^11^ viral particles	Intracardiac injection at two different locations of peri‐infarcted region	‐	improved on day 14 but deteriorated by day 28	‐	26
		AAV‐miR‐1825	gelatin and silicate gel	10 μL Gel + 10 μL viral particles, 2 × 10^9^ viral particles		‐	28 d	‐	
miR‐34a	mice with MI injury	LNA‐based miR‐34a		5 mg/kg	Tail‐vein injection 6 h and 2 d after MI	more than 7 d	7 d	‐	60
miR‐99/100 and Let‐7a/c	Mice with MI injury	anti‐miRs‐99/100 and Let‐7a/c coding lentiviruses		1 × 10^11^ viral genome particles	Single intracardiac injection bordering the infarct zone	less than 15 d	15 d	‐	62
		AAV9‐anti‐miR‐99/100 AAV9‐anti‐Let‐7a/c				more than 90 d	90 d	‐	

## 4 CONCLUSION

The limited capacity of the adult CMs to regenerate after cardiac injury is the major obstacle for heart repair. Recently, non‐coding RNAs are emerging as a promising player in boosting cardiac proliferation and regeneration in heart diseases. In this review, we discuss recent non‐coding RNAs associated with cardiac proliferation and potential therapeutic potential. Due to CM proliferation is transient and rare, more precise and powerful tools, such as lineage tracing strategy,^5^, ^24^ would be useful for dynamically capturing the CMs proliferation events and better understanding the mechanism for the regeneration field. In addition, the barrier between basic research and clinical implication requires more effort to overcome. RNA‐based gene therapy works well, however, there are many barriers and limitations, such as pharmacokinetics and pharmacodynamics, hamper the progress. New delivery system, such as extracellular vesicles^90^ and exosomes,^91^, ^92^ and synthetic hydrogel, would improve RNA‐based therapeutic potential. There are tremendous non‐coding RNAs that haven't been annotated, therefore, the perspective on cardiac regeneration stimulated by non‐coding RNAs, advances us deeper understanding the world of non‐coding RNA and novel clinical therapeutic strategies for heart diseases.

## CONFLICTS OF INTEREST

The authors declare no competing interests.

## AUTHOR CONTRIBUTIONS

**Xiaoxuan Dong:** Writing‐original draft (lead). **Xiuyun Dong:** Writing‐review & editing (equal). **Feng Gao:** Writing‐original draft (supporting). **Ning Liu:** Writing‐original draft (supporting). **Tian Liang:** Writing‐original draft (supporting). **Feng Zhang:** Writing‐original draft (supporting). **Xuyang Fu:** Writing‐original draft (supporting). **linbin Pu:** Writing‐original draft (supporting). **Jinghai Chen:** Supervision (equal); Writing‐original draft (lead); Writing‐review & editing (lead).
